# Cascaded collimator for atomic beams traveling in planar silicon devices

**DOI:** 10.1038/s41467-019-09647-3

**Published:** 2019-04-23

**Authors:** Chao Li, Xiao Chai, Bochao Wei, Jeremy Yang, Anosh Daruwalla, Farrokh Ayazi, C. Raman

**Affiliations:** 1 School of Physics, Georgia Institute of Technology, 837 State St, Atlanta, GA 30332 USA; 2 School of Electrical and Computer Engineering, Georgia Institute of Technology, 777 Atlantic Drive NW, Atlanta, GA 30332 USA

**Keywords:** Surface patterning, Lithography

## Abstract

Micro- and increasingly, nano-fabrication have enabled the miniaturization of atomic devices, from vapor cells to atom chips for Bose-Einstein condensation. Here we present microfabricated planar devices for thermal atomic beams. Etched microchannels were used to create highly collimated, continuous rubidium atom beams traveling parallel to a silicon wafer surface. Precise, lithographic definition of the guiding channels allowed for shaping and tailoring the velocity distributions in ways not possible using conventional machining. Multiple miniature beams with individually prescribed geometries were created, including collimated, focusing and diverging outputs. A “cascaded” collimator was realized with 40 times greater purity than conventional collimators. These localized, miniature atom beam sources can be a valuable resource for a number of quantum technologies, including atom interferometers, clocks, Rydberg atoms, and hybrid atom-nanophotonic systems, as well as enabling controlled studies of atom-surface interactions at the nanometer scale.

## Abstract (web summary)

Bringing atomic beam technology to the chip scale is challenging due to the long distance required to filter the velocity distribution. Here, the authors report an engineering strategy for on-chip filtering of the velocity profile of atomic beams by fabricating planar, etched microchannel arrays.

## Introduction

Microfabrication is increasingly making its mark on atomic physics, from atom chips for Bose-Einstein condensation (BEC)^1,2^ to hybrid atom-MEMS systems^3^ and miniature alkali vapor cell clocks and magnetometers^4,5^. Trapping atoms close to surfaces on a chip offers the opportunity to probe fundamental interactions such as the Casimir force^6–8^ that are important at the nanoscale^9–13^, and to realize unique atom-surface probes^14^. For precise force measurements on atoms, continuous atomic beams offer many advantages over trapped atoms and BECs. They are simpler and require less overhead, offer a continuous readout with no dead time between measurements, and potentially have greater signal-to-noise ratio due to a higher average flux. The main drawback is the mismatch in size between a several meter long highly collimated atomic beam apparatus and the nanometer length scales relevant for interactions with surfaces, which makes alignment of source and target challenging. In spite of this, many precise force measurements have been performed using continuous beams, from pioneering studies of Casimir-Polder^15^ and van der Waals forces^16^ to rotation sensing^17,18^ and diffraction from nanometer sized structures^19–21^.

In this work we propose and demonstrate an alternate paradigm, a miniature atomic beam. We inject rubidium atoms directly onto a silicon chip at the source, which eliminates much of the complexity associated with free space transport of atoms to the surface from a magneto-optical trap (MOT) or BEC. This also ensures pinpoint accuracy of beam alignment with chip-scale lithographic features of micron size and smaller that are downstream of the source, thereby addressing the limitations in the free space atomic beam experiments described above. In our approach we route atoms through planar atomic devices, analogous to how optical beams can be routed in-plane on a chip. Beam deceleration and/or cooling, atom interaction with surfaces or other quantum sensing protocols such as atom interferometry, followed by atom detection can all be performed using elements fabricated directly onto the chip surface. This approach also enables the integration of required heaters and other control electronics, and provides a pathway to mass manufacture of atomic devices. An external envelope can provide vacuum levels that are tailored to the application and can often be in the high-vacuum, rather than ultra-high vacuum level.

We use this approach to demonstrate a nontrivial element–a continuous atomic beam whose velocity distribution is tailored by photolithography. We also demonstrate an atomic device that is uniquely enabled by lithography–a “cascaded” collimated atomic beam. The angular distribution of this beam is 40 times purer than that obtained from a traditional collimator, and is produced entirely on-chip within a 3 mm distance. This demonstration of a microfabricated planar device for thermal atomic beams is the first step toward a fully planar continuous atomic beam sensor.

## Results

### Collimator design

A typical approach to free space atomic beam generation uses an array of capillaries connected on one end to a high density atomic vapor^22–25^. These are usually fabricated by bundling together metal or drawn glass tubes, with a large aspect ratio *l*/*d* between the length of each tube *l* and its diameter *d*. Collimation is achieved by limiting the divergence angle HWHM (half-width at half-maximum of the flux angular distribution) *θ*_1/2_, roughly equal to 0.8*d*/*l*^23^. However, this angle is the only adjustable parameter, offering limited control. Moreover, the 3-dimensional nature of a tube bundle makes addressing atoms within individual tubes difficult, as would be needed for some quantum technological applications.

**Fig. 1:**
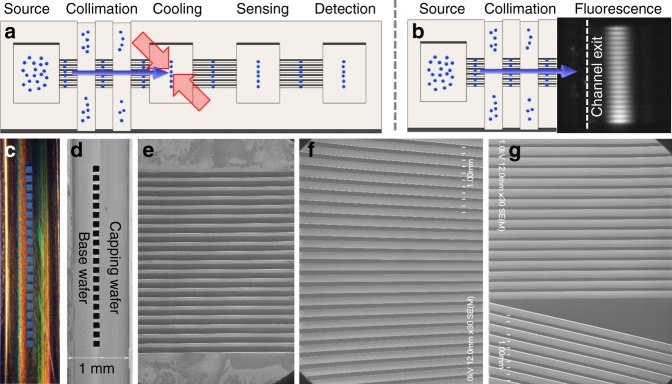
The planar concept for atomic beams. **a** In a fully planar vision, atoms propagate from a source region into a planar device that has been lithographically etched into a silicon chip. Shown schematically are multiple sequential operations including beam formation by collimation, laser deceleration and/or cooling, atom interferometry or other sensing protocols using guided atoms, followed by detection. Blue arrows indicate the direction of the atomic beam propagation. **b** Rubidium atom beam collimation as the first nontrivial element demonstrated in this work. Adjacent image shows the experimentally observed fluorescence output of 20 individually resolved collimation channels. **c** Optical and **d** scanning electron micrograph (SEM) end images of the channels show the etched base wafer and sealing capping wafer. **e**–**g** SEM top images before bonding the capping wafer showing microchannels that (**e**) collimated the atomic beam, **f** produced a focusing beam, and **g** created two beams propagating at a relative angle of 12 degrees. Channel dimensions in **e** were 100 × 10 × 3 mm (*h* × *w* × *l*) dimension, with 50 *μ*m wall thickness between channels. Wall thicknesses as small as 10 *μ*m were fabricated. In all cases high flux, effusive Rb atomic beams were successfully generated

In this work we fabricated planar collimation arrays in silicon, which allows for greater flexibility and control over the array elements, which can also be individually addressed. Fig. 1b shows a top view fluorescence image of 20 individually visible atomic beams generated using this method, each of which has a 100 × 100 *μ*m (*h* × *w*) square cross-section. These beams were created from 20 lithographically defined channels of 3 mm length and the cross-section above, etched into a silicon substrate, as shown in Fig. 1c–e. A second, capping wafer bonded to the top of the channels provided a sealed one-dimensional array with aspect ratio *l*/*w* = *l*/*h* = 30. One end of the array was connected to a rubidium source, and the output flux of the channels was probed on the other end in vacuum using free space fluorescence detection. To demonstrate this flexibility, we generated collimated atomic beam outputs as well as focusing and two-beam outputs. Scanning electron microscope images of these channels without capping wafer bonding are shown in Fig. 1e–g. To our knowledge a two-beam source has never been demonstrated previously.

Unlike conventional machining and/or drawing, the use of photolithography to define the atomic paths allows for a great deal of customization and control with a high spatial resolution less than 1 *μ*m. For example, downstream alignment of source and target become very straightforward, with misalignments less than 10^−5^ radians over a 10 cm wafer length easy to accomplish. This is comparable to the most sensitive atomic beam interferometers that require several meters of length to separate the two interferometer arms^16,18^.

### Cascaded collimator study

**Fig. 2:**
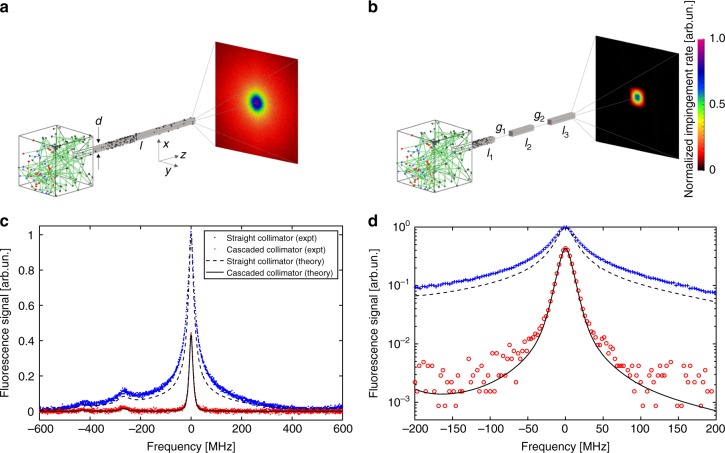
Cascaded collimator concept for high purity atom beam generation. **a** Directed atomic beams generated by a collimating tube, one end of which is connected to a high density atomic vapor comprising an effusive emission source. Two-dimensional image shows the output flux angular distribution from Monte–Carlo simulations as an impingent rate on a downstream screen. **b** A cascaded series of shorter tubes with the same on-axis beam brightness but greatly suppressed off-axis contributions. **c** Measured atomic fluorescence spectra versus excitation laser frequency showing the transverse Doppler distribution of the atomic beam. Peaks at 0, −267, and −424 MHz correspond to transitions between the hyperfine *F* = 2 ground level and *F* = 3, 2, 1 excited levels in ^87^Rb, respectively. Shown are ordinary collimator (blue) and cascaded collimator (red). Solid and dashed lines are corresponding theoretical calculations. **d** Expanded view of ±200 MHz region near the 2–3 peak on a logarithmic scale shows more than a factor of 70 suppression of the wings for the cascaded collimator. Each data point is an average of 5 points in **c**

Using the above lithographic approach, we achieved the “cascaded collimator” that is shown in Fig. 2, and which dramatically exemplifies the customization that is possible. This collimator addresses a major limitation of capillary beams, which is a long “tail” in the angular distribution function extending to large angles $\theta _{1/2} \ll \theta \le \frac{\pi }{2}$^22^. Figure 2a shows Monte–Carlo simulations of a single tube collimator with *l*/*d* = 30 using the Molflow + package, which operates in the molecular flow regime^26,27^. The 2D images are the output angular flux distribution. There is a sharp peak near *θ* = 0 (the center of the image), with a half-width of *θ*_1/2_ that is ~1.8 deg. However, the images show that a significant, low level flux is emitted into large angles, and therefore the width of the central peak does not describe the angular distribution very accurately. In fact, about 99% of the emitted flux is in this broad background with *θ* > *θ*_1/2_. For comparison, the Doppler shift associated with the D2 line of Rb at 780 nm, with its natural linewidth of Γ = 2*π* × 6 MHz, results in a half-width of $\theta _D = \frac{{\Gamma \lambda }}{{4\pi \bar v}} = 0.45$ deg. Only 0.1% of the atoms would thus be excited by a narrow linewidth laser addressing this transition. Many applications, including atomic Raman interferometry, utilize an even narrower slice of forward moving atoms with *θ* < 10^−2^ deg^18^. Thus in many cases of interest the large background ≥99% is a major nuisance.

Figure 2b presents a solution to this problem—a cascaded series of shorter tubes in series with one another. Such a device would be difficult to form using machined collimators due to the need to carefully align each stage to the next with micrometer precision. However, for planar devices it is straightforward to fabricate lithographically by either etching relief regions within the base wafer, or by cutting such reliefs into the capping wafer. In the present work we used the latter method (see Methods). If the overall length and individual tube diameters are the same as for the single tube, then the on-axis beam flux will be unaffected by the cascade. However, atoms that strike the tube walls can now exit the array in the space between successive tubes, i.e., they can be removed within the source itself, which greatly reduces the off-axis emission. The key innovation of this device is that an atom, which enters a gap region is more likely to leave immediately rather than passing all the way down the subsequent tube. If *x* is the relative likelihood of exiting versus propagating through the next tube, then *x* > 1 can be ensured by having a large enough aspect ratio for the individual tubes, e.g., ~10:1. Thus if *W*_0_ is the probability for an atom to pass through a single tube of length *L*, then a simple geometrical argument shows that the probability *W* to pass through a cascade of *n* smaller tubes of length *l* = *L*/(2*n* − 1) is reduced by a factor *W*/*W*_0_ = (2*n* − 1)/*x*^*n*−1^ (see Supplementary Note 1 for estimates). In our experiment the vapor in adjacent gaps is not truly isolated, preventing us from achieving this exponential scaling with *n*. Nonetheless, we achieved a substantial degree of suppression *W*/*W*_0_ = 0.024 ≈ 1/40 for *n* = 3, a number consistent with Monte–Carlo simulations performed on our actual geometry. Further improvements could be made by engineering better vapor isolation through direct, on-chip pumping methods^28^, and the theoretical limit for our estimated value of *x* ~50 is around 10^−3^.

We used Doppler-sensitive laser spectroscopy on the Rb D2 line to experimentally demonstrate this effect. With this technique we could determine the transverse velocity (*v*_⊥_) distribution of the atomic beams generated by these collimators (see Methods). From these data for small angles we could infer their angular distribution through a straightforward transformation ${\mathrm{sin}}\theta = v_ \bot /\bar v$, where $\bar v \approx 300\,{\mathrm{m}}/{\mathrm{s}}$ is the mean velocity of ^87^Rb atoms when oven temperature equals 100 C. The spectral data are shown in Fig. 2c for both a single long tube as well as a cascade of *n* = 3 tubes. Multiple hyperfine resonances between the ^87^Rb 5*S*_1/2_, *F* = 2 ground state and the 5P_3/2_, *F*′ = 1,2,3 levels appeared as narrow peaks as a function of the probe frequency. The strongest such peak is the *F*′ = 3 level, centered at zero frequency offset. The natural linewidth of this transition is 6 MHz^29^, while our laser linewidth is less than 1 MHz. For the single straight collimator this peak has a narrow FWHM of 42 MHz, but contains broad wings visible up to 400 MHz detuning. For comparison, atoms coming out from the nozzle at 45° have an approximate Doppler shift of $\bar v\,{\mathrm{sin}}\,45^{\circ}/\lambda = 270\,{\mathrm{MHz}}$ showing that many atoms propagate at a very large angle to the main beam axis with this collimator.

**Fig. 3:**
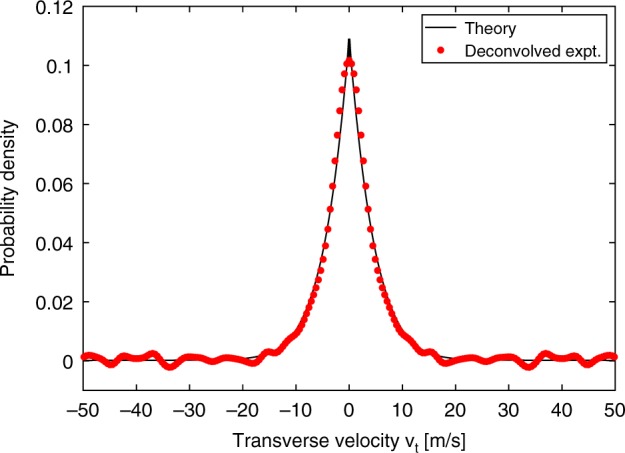
Narrow angular spread of the cascaded collimator. Transverse velocity distribution derived from the measurement by deconvolution, along with the theoretical prediction from Monte–Carlo simulations

**Fig. 4:**
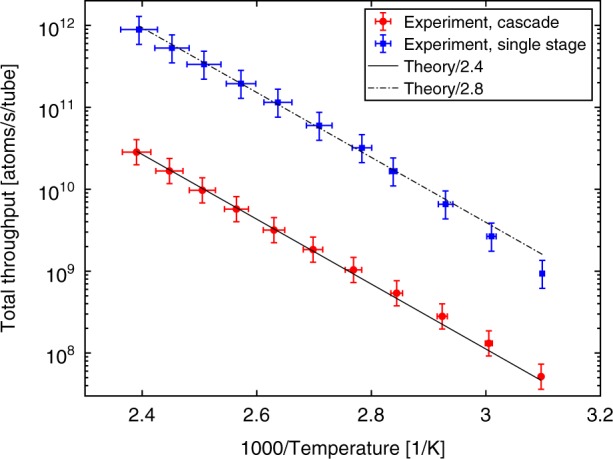
Output flux of the planar atomic beam. Measured throughput of the planar atomic beam for both the ordinary and cascaded collimator geometries confirms the factor of 40 suppression of total flux in the latter case. Data are in good agreement with the prediction of an effusive flux model based on the known Rb vapor pressure. The model throughput has been reduced by a factor of 2.4 (2.8) for the cascaded (ordinary) collimators. Horizontal error bars reflect temperature variation across the oven, while vertical error bars reflect both calibration errors and uncertainty in determination of peak fluorescence

By comparison, the data for a cascaded collimator with *n* = 3 tubes is also shown in Fig. 2c. It has an even narrower FWHM of 18 MHz indicating superior collimation, and the tail of the distribution has been completely suppressed. Both data sets were taken at the same source temperature of 100 C as well as identical illumination conditions (probe laser power and beam waist), so the height of the spectral peaks can be directly compared between the two collimators subject to a temperature uncertainty of ±5C between the two ovens. That is, the peak height accurately reflects the actual number of atoms entering the probe laser volume in both cases. It was larger by a factor of 2.5 for the straight collimator due to the one-dimensional nature of the spectrum, which only differentiates atoms based on their velocity *v*_*y*_ along the laser propagation $(\hat y)$ direction. Atoms with a finite velocity *v*_*x*_ and the same value of *v*_*y*_ are counted in the spectrum equally. For the straight collimator there are many more such atoms compared with the cascaded collimator, which explains the larger peak signal for the former. Using the deconvolved angular distribution and the total throughput (see Figs. 3 and 4), we estimate that the on-axis brightness of both collimators is identical^22–24^. Therefore, while it suppresses the off-axis flux, the cascaded collimator does not reduce the on-axis flux.

For future applications employing miniature atomic devices we quantified the degree of beam purity that was achieved in our setup. To this end we have plotted the same data on a log scale in Fig. 2d within a more narrow frequency range ±200 MHz of the 2–3 transition. The fluorescence signal could be observed with nearly 3 decades of dynamic range by carefully subtracting out backgrounds (see Methods). The resulting noise floor was 170 *μ*V_*rms*_, which at saturation parameter of 1.2 corresponds to a mere ≈100 detected atoms on-resonance. The solid and dashed lines represent theoretical predictions for the spectral distribution of fluorescence. The theoretical curves combined the Molflow+ angular distributions with a master equation solution to the probe laser interaction with a ^87^Rb atom inclusive of hyperfine and Zeeman sub-levels (see Supplementary Note 3). The agreement between predicted and measured spectra is excellent, confirming the high degree of purity of the cascaded collimator beam. Apart from the peak height, the theory has no adjustable parameters. It underestimates the off-axis flux for the straight collimator by a factor of 1.4 due to the one-dimensional, rather than two-dimensional nature of the theoretical calculation. No systematic discrepancy was found in the case of the cascaded collimator, with the slight excess noise at large detunings caused mainly by digitizer noise in the oscilloscope as well as photodiode dark noise. Our experimental data in Fig. 2d show that the wings of the distribution at 200 MHz detuning, corresponding to atoms moving at an angle of 30 degrees from the main axis, are reduced by a factor more than 70.

The measured FWHM of 18 MHz was not much larger than the power-broadened natural linewidth 9 MHz of the transition. Using a deconvolution procedure, we estimated that the transverse Doppler broadening had a HWHM = 4 m/s, which implies a very narrow beam divergence angle *θ*_1/2_ = 0.013 rad. Broadening due to laser frequency noise and Zeeman shifts were at the 1 MHz level. Such a well-collimated beam is remarkable given its miniature size. To our knowledge this is the smallest atomic beam that has ever been realized with such collimation properties. Free space collimators require a clean-up chamber with 10 s of centimeter length to achieve the same beam divergence and suppression of off-axis backgrounds. These experimental data demonstrate the power of microfabrication. Figure 3 shows the deconvolved transverse velocity distribution as well as the Monte–Carlo prediction, with very good agreement observed between the two (procedure described in Supplementary Note 4). The velocity distribution has a sharp cusp at zero, dropping off rapidly (the deconvolution process resulted in unphysical oscillations in the wings of the data). Unlike the straight collimator, there is no long tail at large velocities, as those atoms have been effectively filtered out by the gap regions between successive tubes.

From the measured peak fluorescence and beam velocity distributions we could determine the overall throughput of the two devices^30^ over a broad range of temperatures from 50 to 150 C corresponding to 3 orders of magnitude in flux. Figure 4 shows the results. It confirms that although the on-axis beam brightness of the two collimators is the same, the cascaded collimator emits ≈40 times fewer atoms. Accounting for both ^85^Rb and ^87^Rb isotopes, we estimate that the peak throughput of this collimator reached 3 × 10^10^ atoms/s per tube. For the 20 tubes used in our design, this corresponds to a total flux up to 6 × 10^11^ atoms/s emitted from an 0.2 mm^2^ area. These data demonstrate that the cascaded collimator can replace many atomic beam experiments requiring a high beam brightness. An effusive model using the known Rb vapor pressure curves^29^ is in good agreement over this temperature range. It overestimates the flux by a factor of 2.4 (2.8) for the cascaded (ordinary) collimator, for which uncertainty in the actual Rb pressure in the oven could be a contributing factor. We note that even larger discrepancies of 3.4 have been reported in earlier works with capillaries^30^. For a future design it would be interesting to incorporate the Rb reservoir on-chip. If such a reservoir had the same overall dimensions as the collimator region (1.2 × 5 × 3 mm) we estimate the lifetime at 100 C operation to be around 4000 h, which is quite reasonable. Recirculation of the Rb released into the gap is an intriguing possibility to achieve even greater longevity.

### Other devices fabricated

**Fig. 5:**
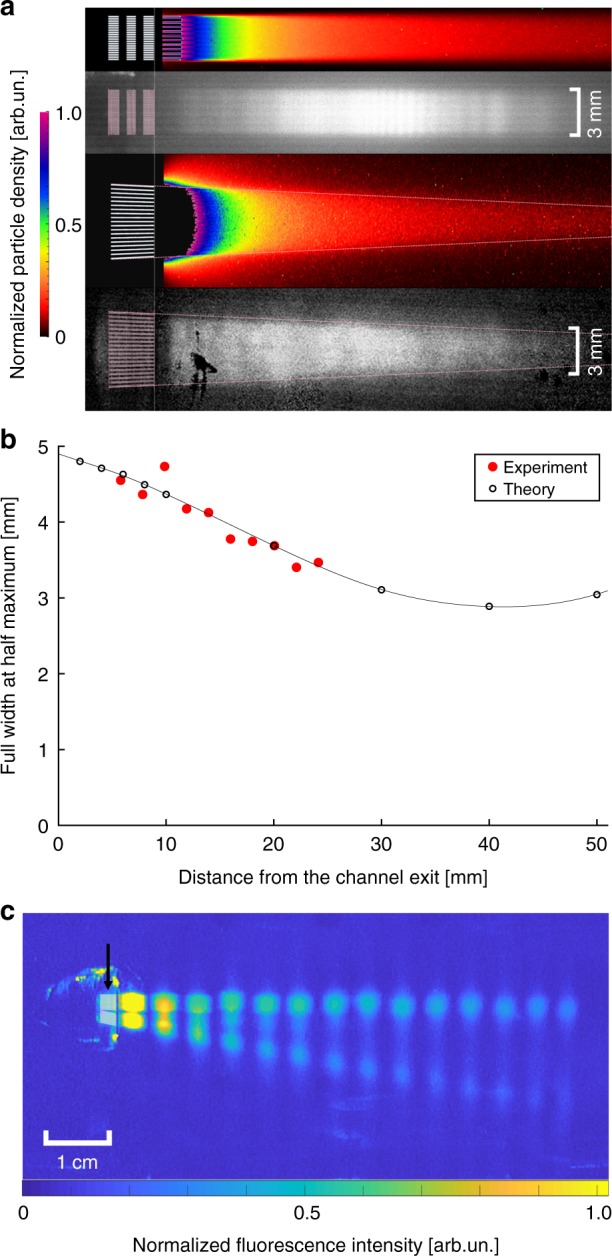
A gallery of tailored atomic beams. From top to bottom are (**a**) cascaded collimator (theory, experiment), and focusing collimator (theory, experiment), **b** measured width of the focusing collimator along with Monte–Carlo theory (solid line), and **c** the two-beam collimator. In **c** the arrow points to the chip location. In **a** the experimental images were obtained by subtracting a background image with the laser tuned away from resonance so no atoms were visible. For the data in **b** the combination of image distortion and pixellation led to a spatial calibration uncertainty of 5% (9%) along the *y*-axis (*x*-axis)

Finally, we show a gallery of experimental data from different lithographically defined collimators in Fig. 5. In all cases we observed high flux, effusive Rb atomic beams from the channels with no indication of clogging or other difficulties. Camera fluorescence images demonstrate the highly tailored velocity distributions achieved by microfabricating the collimator output. Corresponding Monte–Carlo simulations of the output particle density are shown in false Fig. 5a^26,27^. The experimental images were taken by illuminating the atomic beam perpendicular to the atomic velocity such that longitudinal Doppler shifts played no role. The laser beam was expanded in size to 40 mm to cover the atomic beam near the channel exit. Due to the inhomogeneous Gaussian profile of the beam, the fluorescence intensity in the images does not directly correspond to atom density, but simply reflects the transverse spatial distribution of atoms along the atomic beam path. For the cascaded collimators this distribution does not change its size, whereas the focusing chip shows narrowing of the beam due to increasing overlap of the outputs of each channel (individual channels were not resolved in these images). To quantify this, we have plotted the FWHM determined from the camera images in Fig. 5b, which shows a decrease by ≃25% over a distance of 20 mm, in good agreement with the Monte–Carlo theory (the predicted focus occurs at 40 mm from the nozzle output).

A third chip (see Figs. 1g and 5c) was used that generated two atom beams propagating at an angle of 12 degrees to one another from left to right. In this false-color image the atomic beam fluorescence was recorded at 14 distinct locations by illumination parallel to the chip surface. A smaller, 3.5 mm diameter laser beam was moved to each location, where two exposures were taken, one for each atomic beam, whose center frequency was adjusted to account for their different transverse Doppler shifts.

## Discussion

In summary, we have demonstrated a cascaded atomic beam collimator in silicon, the first element in a fully planar atomic device for integrated atomic quantum technologies. These sources could find multiple applications in quantum information processing and entanglement generation in a platform that does not require laser cooling, a considerable simplification. For example, they could be used to couple atomic Rydberg states to microwave cavities, a protocol that has allowed for the generation of unique quantum states^31–34^. Scalable quantum computation models involving multiple such atomic beams have been proposed^35^, for which our chip-scale approach is highly suited. Similarly, Rydberg blockade using miniature alkali vapor cells appears to be a promising route to chip-scale single photon generation for quantum communication applications^36,37^. Such approaches typically require short (nanosecond) laser pulses in order to excite the entire Doppler distribution present in a vapor cell. Atomic beams, by contrast, have the advantage of narrow, Doppler free excitation lines and longer coherence times in the microsecond regime or even longer.

Future work could explore Ramsey excitation in separate, lithographically defined zones for miniature clock applications^38^ or on-chip matter wave interferometry in an architecture similar to ref. ^18^.  The 4 m/s transverse velocity from the cascaded collimator would allow transverse laser cooling within 3 cm^39^, which could enable loading of atoms into optical and/or magnetic waveguides on-chip in a compact geometry^2^. Stimulated forces are an intriguing future direction for centimeter length beam deceleration to directly load a chip MOT^40–42^. Silicon collimators may also prove useful for alkaline earth or other atom sources that require a high operating temperature above 400 C. Finally, we note that multiple wafers can be stacked together using a multi-stack wafer bonding technique^43^ to realize a larger array with greater flux while maintaining on-chip addressability of the individual channels.

## Methods

### Fabrication

**Fig. 6:**
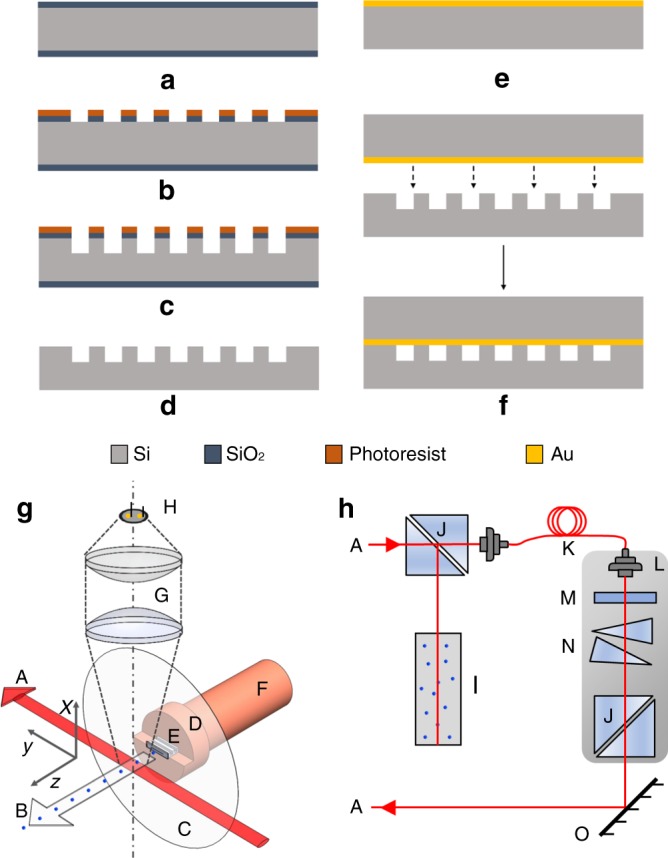
Fabrication and testing procedure. **a** Grow Oxide (**b**) Pattern PR and Etch Oxide **c** Silicon DRIE (**d**) Strip PR/oxide mask (**e**) Evaporate Au (**f**) Si/Au Eutectic Bonding. **g** atom beam production; **h** laser frequency and beam control. A: laser beam. B: atom beam. C: shim stock. D: nozzle holder. E: silicon chip collimator. F: copper tube. G: plano-convex lenses, f = 75 mm. H: photodiode. I: rubidium cell for saturation spectroscopy. J: polarizing beamsplitter. K: optical fiber and fiber coupler. L: translational stage. M: *λ*/2 waveplate. N: prism pair. O: mirror

The fabrication process (see Fig. 6) of these collimators includes two silicon wafers bonded together to form an enclosed structure. First, about 2 *μ*m of oxide was grown on the base wafer (a). Then, about 3 *μ*m of positive photoresist was patterned and the oxide was etched in an RIE process, which formed the mask for the collimators (b). After the oxide etching, the collimators were etched using the Bosch DRIE process up to the required depth of 100 *μ*m (c). The wafer was then cleaned and the remaining oxide was etched away in 49% HF solution to form the completed base wafer (d). Gold was evaporated on the capping wafer (e). The two wafers were then bonded together using a Si–Au eutectic bond at 450 C. Finally, the different dies were diced across the wafer according to the length of the collimators, to give us a completely sealed structure, with access to the two ends of the collimator lengths (f). The cascaded collimator was realized by partially dicing through the bonded wafers in two places, thus breaking the seal (see Supplementary Fig. 1). This resulted in channel lengths of *l*_1_, *l*_2_, *l*_3_ = 690, 610, 660 *μ*m, while the diced region widths were *g*_1_, *g*_2_ = 510, 500 *μ*m.

The Rb oven is of a standard design involving a pinched copper tube (3/8 inch OD, 6 inch length) containing a 200 mg Rb ampoule. The silicon chip collimator is glued into a slit at the center of a separately heated nozzle holder using thermally conductive epoxy (see Fig. 6g). A temperature difference anywhere between 8–16 Celsius was maintained between the nozzle and the oven body. For the cascaded collimator, a piece of shim stock placed directly at the exit face of the silicon chip was used to separate the released off-axis vapor from the forward atom beam. The oven temperature was recorded using thermistors and controlled using external heaters of a standard variety.

### Measurement

We performed Doppler-sensitive laser spectroscopy of the atomic beams using a free-running diode laser (Toptica DLX110) scanned at a frequency of 5 Hz, with scan range ≲1 GHz. We recorded fluorescence induced by a probe laser exciting the ^87^Rb D2 resonance near *λ* = 780 nm, and propagating perpendicular to the atomic beam, as in Fig. 6g. Thus Doppler shifts due to the Maxwell-Boltzmann distribution of longitudinal velocities were eliminated. Several hyperfine resonances between the 5*S*_1/2_, *F* = 2 ground state and the 5 P_3/2_, *F*′ = 1, 2, 3 levels were detected as a function of the probe frequency. The width of each peak was Doppler broadened due to the transverse velocity distribution of the emitted atoms, and the fluorescence intensity measured this distribution.

The probe light was transferred from an optical table for the laser frequency control to a table for the atomic beam experiment through a single mode polarization-maintaining fiber. The fiber output, a half-wave plate, a prism pair and a polarizing beamsplitter are coaxially aligned and mounted together onto a single vertical translation mount (see Fig. 6h), so that we could precisely scan the laser beam in the vertical dimension through tweaking the adjustment screw for the base plate. In this way, we can better locate the axial part of the atomic beam and vertically walk the laser beam without changing its parallelism to the plane spanned by our planar array structure. Light coming out from the fiber is horizontally expanded by the prism pair, p-polarized by the beamsplitter, and then reflected by a broadband dielectric mirror, after which it enters the vacuum region through a standard low-iron glass viewing window (6 × 6 inches). Two cage rods along the laser beam mount the mirror and allow the mirror to move along the laser beam without changing the laser-atom beam orthogonality. Therefore, we could capture a sequence of camera images along the atom beam by scanning the mirror position without introducing unwanted Doppler shift (see Fig. 5c).

Atomic fluorescence from the laser spot was collected by two f = 75 mm 2 inch diameter plano-convex lenses and then focused onto a photodiode (Thorlabs DET100A2) covered with a 780 nm interference filter to block room light. We could determine the distance between the center of the laser beam and the nozzle exit through replacing the photodiode by a camera and imaging both the fluorescence spot and the edge (with well-defined 5 mm width) of the silicon chip. Through maximizing the peak height and minimizing the full width half maximum of the fluorescence spectrum, an orthogonal crossing interaction region is achieved. The fluorescence is collected from a location only 6 mm away from the nozzle exit in order to make sure the photodiode sees atoms coming out from all angles. The Gaussian radius (1/*e*^2^) of the laser beam measured at the interaction region is 1.4 mm and 0.5 mm for the horizontal and vertical dimension, respectively. An optical cage system located right above the chamber window mounts the two lenses and the photodiode along the vertical axis of the fluorescence spot when the laser frequency is on-resonance.

The photocurrent is amplified by a preamplifier (DL Model 1211, Gain either 10^8^ or 10^9^ V/A) realizing a rise time of 0.04 or 0.25 ms, respectively. This is much shorter than the minimal signal rise time of 10 ms. No circuit delay broadening is expected and we verify this by monitoring the width of the fluorescence spectrum while varying the scan frequency of the laser from 5 to 100 Hz at Gain = 10^8^ V/A. Both the saturated spectroscopy from the rubidium reference cell for the frequency calibration and the fluorescence spectrum from the atomic beam are recorded and averaged over 64 traces by an oscilloscope (TDS2024C). Background scans were taken 9.5 mm directly below the atomic beam in order to subtract off fluorescence from a weak Rb vapor in the chamber. As an example, for the cascaded collimator data in Fig. 2c, d, the vapor component was 380 *μ*V_*rms*_, or 2.4% of the peak signal. After canceling this background, the residual noise floor for our setup was 170 *μ*V_*rms*_, corresponding to ≈100 on-resonance atoms detected at saturation parameter of 1.2. Measured photocurrent was converted to atomic flux using the calibration procedure described in Supplementary Note 5. The Supplementary Information also describes the theoretical predictions.

## Supplementary information

Supplementary Information

## Data Availability

The data that support the findings of this study, and all code used to generate the theoretical curves for the data in Figs. 2–5 are available from the corresponding author upon reasonable request.

## Code availability

The code used to generate the theoretical curves for the data in Figs. 2–5 are available from the corresponding author upon reasonable request.
