# Supplementary material for: Cascaded collimator for atomic beams traveling in planar silicon devices
Source: Nat Commun. 2019 Apr 23;10:1831. doi: 10.1038/s41467-019-09647-3 (PMC6478944; doi:10.1038/s41467-019-09647-3)
Supplement: Supplementary file 1 — Supplementary Information [file 41467_2019_9647_MOESM1_ESM.pdf]

Supplementary Information for:
Cascaded collimator for atomic beams traveling in planar silicon devices

Chao Li,[1] Xiao Chai,[1] Bochao Wei,[1] Jeremy Yang,[2]
Anosh Daruwalla,[2] Farrokh Ayazi,[2] and C. Raman[1]

[1]*School of Physics, Georgia Institute of Technology,*
837 State St, Atlanta, Georgia 30332, USA

[2]*School of Electrical and Computer Engineering, Georgia Institute of Technology,*
777 Atlantic Drive NW, Atlanta, Georgia 30332, USA

Supplementary Note 1: Simplified model of cascaded collimator transmission

In our work we utilized Monte Carlo simulations and an actual CAD model of the cascaded collimator in order to obtain the most accurate theoretical predictions. However, it is also useful to compare these with a simple analytical model for the cascaded collimator transmission probability W. Supplementary Figure 1a shows an equivalent circuit that represents the vacuum resistance $R = 1/C$, where C is the vacuum conductance [1]. Using this circuit we can calculate W using only ratios of conductances, and not absolute values, with pre-factors proportional to the mean atomic velocity cancelling out.

For a single tube of diameter d and length l, $C \propto AW_l$, where $A = \frac{\pi}{4}d^2$ is the cross-sectional area and $W_l \approx \frac{4d}{3l}$ is the Clausing factor for a single tube in the limit $l/d \gg 1$ [2]. W is the probability that an atom which enters the tube on one end will exit the tube on the other end. Defining $V \equiv P$ as the potential, where P is the pressure and $I \equiv Q$ as the current, where Q is the particle flux, a cascade of n tubes separated by $n-1$ gaps is shown in Supplementary Figure 1a. As defined in the main text, $x = R/R_s$ is the relative likelihood that an atom which enters a gap region prefers to leave immediately (via R_s) rather than passing through the subsequent tube R in the network. If $x \gg 1$ then the potential at the kth node of the network is $V_k \approx \frac{V_{k-1}}{x}$. The current through the final resistor $I = \frac{V_{n-1}}{R} = \frac{V_0}{Rx^{n-1}}$. Comparing this to a single tube, $I = (2n-1)V_0/R$ since R is proportional to the tube length and $L/l = 2n-1$. Thus $W/W_0 = \frac{2n-1}{x^{n-1}}$.

An order of magnitude approximation can be made for the above, referring to the geometry in Supplementary Figure 1b. The resistance of an isolated rectangular tube of cross-sectional area $A = w \times h = \frac{\pi}{4}d^2$ is $\propto \frac{l}{d^3}$, where d, l are the tube effective diameter and length. Ignoring factors of order unity, in the presence of an array of m tubes in parallel, R is reduced by a factor $m = \frac{fb}{d}$, where b is the transverse width of the array and f the open fraction. Thus $R \propto \frac{l}{d^2 fb}$. Assuming $a \ll b$ as appropriate to a thin wafer, and assuming both $a, c \sim l \ll b$, then most atoms exit the gap at the top, and not its sides. Thus the resistance $R_s \propto \frac{1}{bc} \approx \frac{1}{bl}$. This gives $x = R/R_s = \frac{l^2}{fd^2}$ independent of the wafer dimensions. For 3 tubes, $f \approx 0.7$, $L = 3$mm, $d = 0.1$mm, $\frac{l^2}{fd^2} \approx 54$, which gives $W/W_0 \approx 2 \times 10^{-3}$. In our experiment the vapor in each gap is not isolated, and thus Monte-Carlo simulations performed for our geometry predict $W/W_0 \simeq 1/40$, an order of magnitude smaller than the ideal case. Supplementary Figure 1c shows an image of the fabricated cascaded collimator.

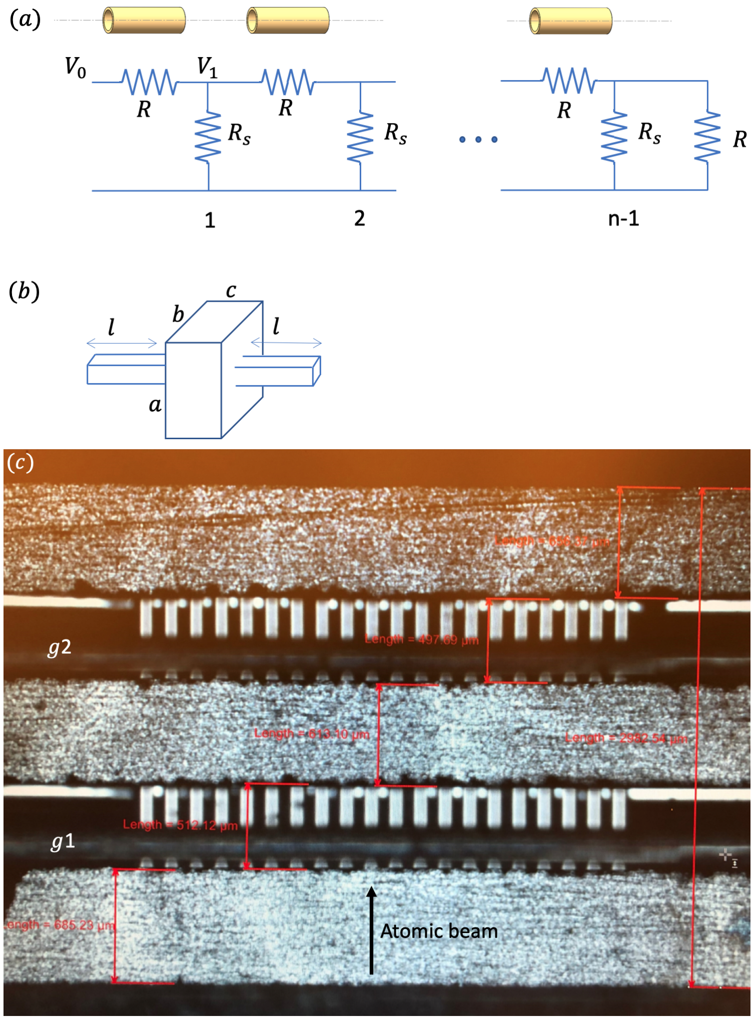

Supplementary Figure 1. (Color Online). Cascaded collimator circuit model and physical implementation. (a) Equivalent circuit model of cascaded collimator, with $1/R$ being the tube vacuum conductance and $1/R_s$ the conductance between the gap regions and the vacuum envelope. (b) Simple model of two rectangular cross-section tubes separated by a gap of dimensions a, b, c that can be used to predict the ratio $x = R/R_s$. (c) Microscope image of cascaded collimator in top view showing the 2 gap regions g_1 and g_2 where the capping wafer was removed. Atoms propagated through the channels in the direction of the arrow. Some of the channel walls are still visible after dicing due to the asymmetry of the dicing blade. The channel lengths were $l_1, l_2, l_3 = 690, 610, 660$ μm, while the diced region widths were $g_1, g_2 = 510, 500$ μm, as defined in Figure 2b of the main text.

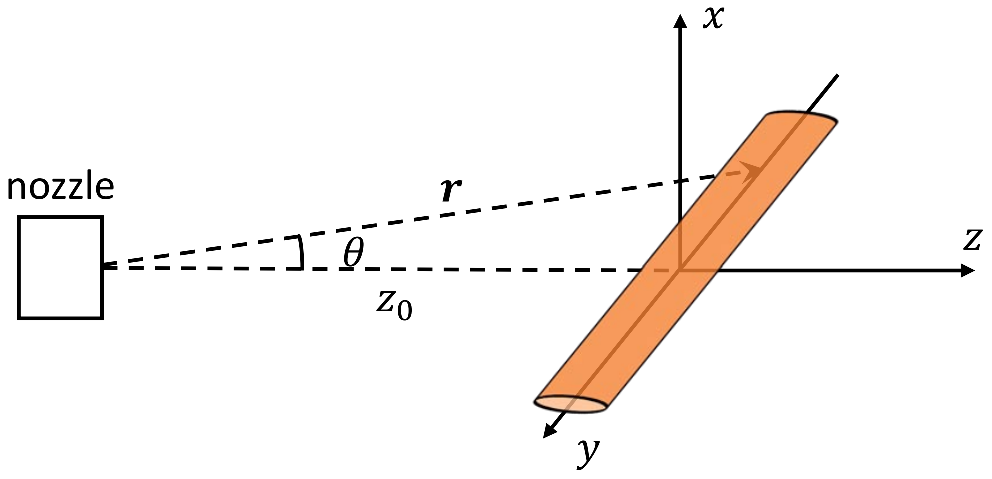

Supplementary Figure 2. Schematics of the experimental setup for detection. The atomic beam propagates along the z axis. The laser beam propagates along the y axis. The orange elliptical cylinder indicates the fluorescence collection volume.

Supplementary Note 2: Total photon scattering rate and atomic density

The quantity that relates our experiment and theory is the total photon scattering rate, which is the number of photons scattered per unit time by atoms within the collection volume. The total photon scattering rate, $\gamma(\omega)$, can be given by [3, 4]

$$\gamma(\omega) = \kappa N_{\mathrm{t}} \int_0^\infty \mathrm{d}v \int_B \mathrm{d}V\, n(\mathbf{r}, v) R_{\mathrm{sc}}(\omega - kv \sin\theta \sin\phi), \tag{1}$$

where $N_{\mathrm{t}} = 20$ is the number of tubes. $\kappa = 27.83\%$ is the abundance of ^{87}Rb atoms. $n(\mathbf{r}, v)\,\mathrm{d}v$ is the number density of the atoms at position $\mathbf{r}$ within the speed range $v \sim v + \mathrm{d}v$, produced by each tube. B is the fluorescence collection volume defined by the laser beam and the collection optics (see Supplementary Figure 2). $R_{\mathrm{sc}}(\omega)$ is the single atom photon scattering rate with laser frequency ω. The factor $(kv \sin\theta \sin\phi)$ comes from the transverse Doppler shift where $k = 2\pi/\lambda$ is the laser wave vector in terms of the optical wavelength $\lambda = 780$ nm. θ, ϕ are polar and azimuthal angles in spherical coordinates.

To relate $n(\mathbf{r}, v)$ to the angular distribution of atomic flux from the collimators, we follow the convention in [2]. The atomic flow from a collimator is given by

$$\mathrm{d}\dot{N} = \frac{\dot{N}}{\pi} f(\theta) F_T(v)\, \mathrm{d}\Omega\, \mathrm{d}v\,, \tag{2}$$

where $\mathrm{d}\dot{N}$ is the number of the atoms with speed in the range $v \sim v + \mathrm{d}v$ entering the solid angle $\mathrm{d}\Omega$, per unit time. $\dot{N}$ is the total throughput per tube. $f(\theta)$ is the angular distribution function. $f(\theta)\,\mathrm{d}\Omega$ is the probability of atoms entering the solid angle $\mathrm{d}\Omega$ with angle θ $(0 \leqslant \theta \leqslant \pi/2)$, which is normalized as $\int_{2\pi} f(\theta)\,\mathrm{d}\Omega = \pi$. $F_T(v)$ is the Maxwell-Boltzmann distribution of particle flux:

$$F_T(v)\,\mathrm{d}v = \frac{2}{\alpha}(\frac{v}{\alpha})^3 \mathrm{e}^{-(v/\alpha)^2}\,\mathrm{d}v\,, \tag{3}$$

with $\alpha = \sqrt{2k_{\mathrm{B}}T/m}$. Here k_{B} is the Boltzmann constant. T is the temperature. m is the atomic mass. $\mathrm{d}\dot{N}$ also represents the number of atoms going through an area $\mathrm{d}S = r^2\,\mathrm{d}\Omega$ per unit time

$$\mathrm{d}\dot{N} = n(\mathbf{r}, v)v\,\mathrm{d}S\,\mathrm{d}v = n(\mathbf{r}, v)vr^2\,\mathrm{d}\Omega\,\mathrm{d}v\,, \tag{4}$$

Comparing Supplementary Equation (2) and Supplementary Equation (4) we have

$$n(\mathbf{r}, v) = \frac{\dot{N}}{\pi}\frac{f(\theta)F_T(v)}{vr^2}. \tag{5}$$

Supplementary Note 3: Theoretical calculation of fluorescence spectrum

We assume $n(\mathbf{r}, v)$ is weakly dependent on x and z within the collection volume (1-D approximation). With this approximation $\gamma(\omega)$ becomes

$$\gamma(\omega) = \pi a b z_0 \kappa N_{\mathrm{t}} \int_0^{\infty} \mathrm{d}v \int_{-\pi/2}^{\pi/2} \mathrm{d}\theta\,(\sec^2\theta) n(\mathbf{r}, v) R_{\mathrm{sc}}(\omega - kv\sin\theta), \tag{6}$$

where a, b are the waists of the elliptical Gaussian laser beam in $\hat{x}$ and $\hat{z}$ directions. z_0 is the distance between the laser beam axis and the nozzle.

Combining Supplementary Equation (6) and Supplementary Equation (5) we have

$$\gamma(\omega) = \frac{ab\kappa N_{\mathrm{t}}\dot{N}}{z_0} \int_{-\frac{\pi}{2}}^{\frac{\pi}{2}} \mathrm{d}\theta \int_0^{\infty} \mathrm{d}v\,\frac{F_T(v)}{v} f(|\theta|) R_{\mathrm{sc}}(\omega - kv\sin\theta). \tag{7}$$

The fluorescence spectrum is proportional to $\gamma(\omega)$ given by Supplementary Equation (7). When the laser frequency is off-resonant from the ^{87}Rb $F = 2 \to F' = 3$ transition, optical pumping to other hyperfine levels makes it difficult to write a closed form for $R_{\mathrm{sc}}(\omega)$. The effective saturation intensity given by $3.054\,\mathrm{mW}\cdot\mathrm{cm}^{-2}$ [5] for linearly polarized light. The laser beam intensity at the center of the beam profile is (3.8 ± 0.1) mW/cm^2. Then the

effective saturation parameter is 1.2, under which the atoms are well saturated. When the laser is coupling the $F = 2 \to F' = 3$ transition, the optical pumping is weak because the $F' = 3 \to F = 1$ spontaneous emission is forbidden. However, when the laser is coupling the $F = 2 \to F' = 2$ or $F = 2 \to F' = 1$ transition, the atoms will be pumped to the $F = 1$ states in $1\,\mu$s. In comparison, the atom needs about $10\,\mu$s to traverse the laser beam profile. Although the coupling strength for all 3 transitions are similar, optical pumping reduces the fluorescence intensities of the $F = 2 \to F' = 1$ and $F = 2 \to F' = 2$ transitions relative to the strong $F = 2 \to F' = 3$ transition.

To include the effect from the optical pumping, we calculated $R_{\mathrm{sc}}(\omega)$ by numerically solving the master equation [6] with the full ^{87}Rb D2 structure:

$$\dot{\rho}(t) = -\frac{i}{\hbar}[H(\mathbf{r}, t), \rho(t)] + \Gamma \left(\frac{2J' + 1}{2J + 1}\right) \sum_{q=-1}^{1} \mathcal{D}[\Sigma_q]\rho(t). \tag{8}$$

^{87}Rb has nuclear spin quantum number $I = 3/2$. The electronic total angular momentum quantum number of ground and excited states for the D2 transition are $J = 1/2$ and $J' = 3/2$. The total angular momentum quantum number of the ground and excited states are $F = 1, 2$ and $F' = 0, 1, 2, 3$. So the Hilbert space has dimension of 24. Here $\rho(t)$ is the 24×24 density matrix describing the atomic internal state. $H(\mathbf{r}, t) = H_{\mathrm{A}} + H_{\mathrm{AF}}(\mathbf{r}, t)$ is the Hamiltonian including both the atomic part and the atom-field interaction. $\mathcal{D}[\Sigma_q]$ is the Lindblad superoperator and Σ_q is the jump operator, which are defined in [6].

In the simulation, the atomic velocity was fixed as the mean velocity $\bar{\mathbf{v}}$ so that the atomic position was $\mathbf{r} = \bar{\mathbf{v}}t$. The Gaussian distribution of the laser intensity entered the simulation by the spatial dependence of the Hamiltonian $H(\mathbf{r}, t)$. The atoms traveled normal to the Gaussian beam. The simulation time was fixed by the transit time of the mean velocity, which was about $10\,\mu$s. The time dependence of the density matrix $\rho_\omega(t)$ under the laser frequency ω was solved by integrating the Supplementary Equation (8). The scattering rate without Doppler shift was given as an integral of the excited states population:

$$R_{\mathrm{sc}}(\omega) \propto \int \mathrm{d}t \sum_{F', M'} \langle F', M' | \, \rho_\omega(t) \, | F', M' \rangle \, . \tag{9}$$

The fluorescence spectrum was then calculated with Supplementary Equation (7), where $f(\theta)$ was given by the Molflow+ simulation [7, 8]. $R_{\mathrm{sc}}(\omega)$ was given by the Supplementary Equation (9). We calculated the spectra for both the cascaded collimator and the ordinary

collimator. The theoretical curves were normalized to the peak values of the experimental data and both were plotted in Figure 2c,2d of the main paper.

Supplementary Note 4: Atomic beam transverse velocity distribution

For the ^{87}Rb $F = 2 \to F' = 3$ transition with linearly polarized light coupling, $R_{\rm sc}(\omega)$ is given by[5]:

$$R_{\rm sc}(\omega) = \alpha \frac{\Gamma}{2} \frac{s}{1 + s + 4\left(\dfrac{\omega - \omega_0}{\Gamma}\right)^2}, \tag{10}$$

where α is the atomic population initially in the $F = 2$ state, which is about 5/8. $\Gamma = 2\pi \times 6.0666\,\text{MHz}$ is the natural linewidth [5]. ω_0 is the resonance frequency. s is the saturation parameter with the effective saturation intensity given by $3.054\,\text{mW} \cdot \text{cm}^{-2}$ [5] for linearly polarized light, calculated at the center of laser beam.

Through the change of variables $v_\perp = v \sin\theta$, we can re-write Supplementary Equation (7) as a convolution of the transverse velocity distribution and the scattering rate

$$\gamma(\omega) = N_B \int_{-\infty}^{\infty} \mathrm{d}v_\perp \, p(v_\perp) R_{\rm sc}(\omega - k v_\perp), \tag{11}$$

where N_B is the atom number in the collection volume. $\int_{-\infty}^{\infty} \mathrm{d}v_\perp \, p(v_\perp) R_{\rm sc}(\omega - k v_\perp)$ is the averaged scattering rate for atoms in the collection volume [4]. $p(v_\perp)$ is the transverse velocity distribution

$$p(v_\perp) = \frac{ab\kappa N_{\rm t} \dot{N}}{N_B z_0} \int_0^{\pi/2} \mathrm{d}\theta \, \frac{f(\theta) F_T(|v_\perp| / \sin\theta)}{|v_\perp|}. \tag{12}$$

It can be verified that $p(v_\perp)$ is normalized as $\int_{-\infty}^{\infty} \mathrm{d}v_\perp \, p(v_\perp) = 1$.

We theoretically calculated $p(v_\perp)$ using Supplementary Equation (12), where $f(\theta)$ was given by the Molflow+ simulation. The experimental result was deconvolved from Supplementary Equation (11) by replacing the left-hand side with the experimental value for $\gamma(\omega)$ and using Fourier transform routine with Tikhonov regularization [9]. We only used the blue detuned half of the spectrum for deconvolution. Both the experimental and the theoretical $p(v_\perp)$ were normalized as $\int_{-\infty}^{\infty} \mathrm{d}v_\perp \, p(v_\perp) = 1$ and plotted in Figure 3 of the main paper.

Supplementary Note 5: Total throughput calibration

The measured voltage through a photodiode sensor and a current amplifier will be the following

$$V_{\text{out}}(\omega) = \hbar\omega_0\gamma(\omega)R_{\text{resp}}G\eta, \tag{13}$$

where $R_{\text{resp}} = 0.6$ A/W is the responsivity of the photodiode, $G = 10^8$ or 10^9 V/A is the gain of the current preamplifier and $\eta = 1.2\%$ is the overall photon collection efficiency that is subjected to the transmission of optics and the solid angle over which the emitted photons could be collected.

The fluorescence spectrum was recorded by measuring V_{out} for different values of laser frequency ω. The experimental value of $\gamma(\omega)$ was then calculated using Supplementary Equation (13). Combining Supplementary Equation (1), Supplementary Equation (5) and Supplementary Equation (13), we have

$$\dot{N} = \frac{V_{\text{out}}(\omega)}{\hbar\omega_0 R_{\text{resp}} G\eta\kappa N_{\text{t}}\zeta}, \tag{14}$$

where ζ is

$$\zeta = \int_0^\infty \mathrm{d}v \int_B \mathrm{d}V\, \frac{f(\theta)F_T(v)}{\pi v r^2} R_{\text{sc}}(s(\mathbf{r}), \omega - kv\sin\theta\sin\phi). \tag{15}$$

For better calibration of the total throughput, we carried out the integral ζ in 3-D space to include the spatial variance of fluorescence, where the integral volume was a cuboid: $-4b \leq x \leq 4b,\ -l/2 \leq y \leq l/2,\ z_0 - 4a \leq z \leq z_0 + 4a$. Here $l = 14.4\,\text{mm}$ is the horizon of the imaging system. z_0 is the position of the laser beam on the z axis. $z_0 = 5.55\,\text{mm}$ for the ordinary collimator experiment and $z_0 = 5.90\,\text{mm}$ for the cascaded collimator experiment.

To include the effects of the spatial dependence of laser intensity, R_{sc} should be rewrite as

$$R_{\text{sc}}(s(\mathbf{r}), \omega) = \alpha\frac{\Gamma}{2}\frac{s(\mathbf{r})}{1 + s(\mathbf{r}) + 4\left(\dfrac{\omega - \omega_0}{\Gamma}\right)^2}, \tag{16}$$

where the spatial dependence of the saturation parameter comes from the Gaussian beam intensity distribution.

The experimental value of $\dot{N}$ was then calculated by Supplementary Equation (14), where we chose $\omega = \omega_0$. $f(\theta)$ was given by the Molflow+ simulation. The measured total throughput was compared with an effusive flux model[2]:

$$\dot{N} = \frac{n\bar{v}}{4}AW, \tag{17}$$

where A is the cross-sectional area of the tubes, W is the Clausing factor determined from the Monte Carlo simulations, and n is the atom number density in the oven determined from the Rb vapor pressure curves [5]. Both the experiment (Supplementary Equation (14)) and theory (Supplementary Equation (17)) curves are plotted in Figure 4 of the main paper.

Supplementary References

[1] O'Hanlon, J. F. *A User's Guide to Vacuum Technology* (Wiley, Hoboken, 2003), 3rd edn.

[2] Beijerinck, H. & Verster, N. Velocity distribution and angular distribution of molecular beams from multichannel arrays. *J. Appl. Phys.* **46**, 2083–2091 (1975).

[3] Demtröder, W. *Laser Spectroscopy* (Springer, Berlin, 2003).

[4] Schioppo, M. *et al.* A compact and efficient strontium oven for laser-cooling experiments. *Rev. Sci. Instrum.* **83**, 103101 (2012).

[5] Steck, D. A. *Rubidium 87 D line data.* Available online at http://steck.us/alkalidata (revision 2.1.5, 13 January 2015).

[6] Steck, D. A. *Quantum and atom optics.* Available online at http://steck.us/teaching (revision 0.11.0, 18 August 2016).

[7] Molflow+ is a Monte Carlo code developed at CERN by R. Kersevan and M. Ady. *website: cern.ch/molflow* .

[8] Kersevan, R. & Pons, J.-L. Introduction to molflow+: New graphical processing unit-based monte carlo code for simulating molecular flows and for calculating angular coefficients in the compute unified device architecture environment. *J. Vac. Sci. Technol. A* **27**, 1017–1023 (2009).

[9] Hansen, P. C. *Rank-deficient and Discrete Ill-posed Problems: Numerical Aspects of Linear Inversion*, vol. 4 (Siam, 2005).
